# A Complex Diagnostic Challenge of Dual Antibiotic-Induced Drug Reaction With Eosinophilia and Systemic Symptoms (DRESS) Syndrome With Multiorgan Involvement

**DOI:** 10.7759/cureus.99714

**Published:** 2025-12-20

**Authors:** Bola Habeb, Christopher Wright, Mina Motakhaveri, Erica S Thomson, Matthew Fowler

**Affiliations:** 1 Internal Medicine, Florida State University College of Medicine/Ascension Sacred Heart, Pensacola, USA

**Keywords:** drug-induced liver injury (dili), drug-induced transaminitis, drug reaction with eosinophilia and systemic symptoms (dress) syndrome, eosinophilia, fever with rash, minocycline reaction, skin biopsy punch, systemic steroids, vancomycin-induced dress syndrome

## Abstract

Drug reaction with eosinophilia and systemic symptoms (DRESS) syndrome is a serious, immune-mediated hypersensitivity condition marked by cutaneous eruptions, fever, hematologic abnormalities, and involvement of multiple organ systems. We report a case of DRESS syndrome precipitated by IV vancomycin and minocycline, presenting with fever, diffuse morbilliform rash, marked eosinophilia, transaminitis, and acute kidney injury. The diagnostic process was challenging because of overlapping features between infectious and autoimmune etiologies, necessitating careful clinical correlation, medication review, and application of the Registry of Severe Cutaneous Adverse Reactions (RegiSCAR) criteria. Early recognition enabled prompt discontinuation of the offending agents and initiation of systemic corticosteroids, resulting in gradual improvement in hepatic and renal function. This case underscores the need to maintain a heightened clinical suspicion for DRESS syndrome in patients treated with high-risk antibiotics. It reinforces the importance of prompt recognition and coordinated multidisciplinary care to reduce the risk of lasting organ injury.

## Introduction

Drug reaction with eosinophilia and systemic symptoms (DRESS) is an uncommon yet potentially life-threatening, idiosyncratic adverse response to certain medications. It often manifests as a widespread skin rash accompanied by fever and hematologic abnormalities, most notably eosinophilia and atypical lymphocytosis. It may progress to involve multiple organs, including the liver, kidneys, lungs, heart, and pancreas [1,2]. The reported incidence of DRESS syndrome is low (generally estimated in the range of approximately 1:1,000-1:10,000 exposures). Still, the true incidence is likely underestimated because of variable clinical presentations, delayed onset after drug exposure (typically two to eight weeks), and inconsistent reporting [1,3].

A wide variety of medications have been implicated in DRESS, with classic culprits including aromatic anticonvulsants (e.g., phenytoin, carbamazepine), allopurinol, sulfonamides, and, more recently, multiple classes of antibiotics, including vancomycin and tetracyclines such as minocycline, which have been increasingly reported as triggers in contemporary series and case reports [1,4,5]. The pathophysiology is complex and incompletely understood; proposed mechanisms encompass a delayed T-cell-mediated (type IV) hypersensitivity to drug or reactive metabolites, genetic predisposition related to specific human leukocyte antigen alleles, impaired drug detoxification pathways with accumulation of reactive metabolites, and viral reactivation (classically human herpesvirus-6, but also human herpesvirus-7, Epstein-Barr virus (EBV), and cytomegalovirus (CMV)) that may amplify immune activation and clinical severity [2,6,7].

Diagnosis remains primarily clinical and can be challenging because DRESS mimics infectious, autoimmune, and other severe cutaneous adverse reactions. Validated diagnostic/scoring systems, most commonly the Registry of Severe Cutaneous Adverse Reactions (RegiSCAR) criteria, assess the timing of drug exposure, characteristic rash, fever, hematologic abnormalities, and evidence of internal organ involvement [1,2,8]. These factors are used to classify cases as “no,” “possible,” “probable,” or “definite” DRESS. Histopathology may be supportive but is not pathognomonic [2]. Common laboratory and imaging findings reflect the organ systems involved: markedly elevated transaminases with clinical hepatitis being among the most frequent and serious complications, and acute kidney injury (AKI) (from interstitial nephritis or other mechanisms), myocarditis, pneumonitis, and hematologic dysfunction occurring less commonly but contributing substantially to morbidity and mortality [1,3,9].

Management centers on prompt recognition, immediate withdrawal of the offending drug(s), and supportive care. For patients with notable organ involvement, systemic corticosteroids remain the primary first-line treatment, although optimal dose, taper duration, and evidence from randomized trials remain limited; steroid-refractory or rapidly progressive cases have been treated with IV immunoglobulin, cyclosporine, mycophenolate, or other immunomodulatory agents, and these options are increasingly discussed in recent reviews and case series [10]. Early multidisciplinary involvement (dermatology, allergy/immunology, hepatology/nephrology, and critical care) is recommended for severe cases to monitor for complications and guide immunosuppression. Relapses can occur during steroid tapering or weeks after apparent recovery, and some patients develop chronic sequelae such as persistent organ dysfunction or autoimmune disease, underscoring the importance of close follow-up [2,10].

Given the expanding recognition of antibiotics such as vancomycin and minocycline as potential triggers and the risk for fulminant hepatic and renal complications, clinicians should remain alert to the possibility of DRESS in patients who develop fever, rash, and systemic abnormalities several weeks after starting new medications. This case report illustrates the diagnostic complexity when two antibiotics are temporally associated with the syndrome. It highlights principles for timely diagnosis, discontinuation of culprit drugs, and institution of appropriate immunomodulatory therapy.

## Case presentation

A 51-year-old woman with a medical history significant for seizure disorder, dyslipidemia, restless leg syndrome, migraine, and gastroparesis, and a recent hospitalization for osteomyelitis requiring ankle arthroscopy with extensive debridement and open talus fracture repair, presented to the emergency department with nausea, vomiting, and a syncopal episode. During her hospitalization five weeks earlier, she had received IV minocycline and was discharged on IV vancomycin and piperacillin-tazobactam.

While receiving IV vancomycin at an infusion center, she experienced a syncopal episode. She also reported a three- to four-day history of subjective fevers, nausea, multiple episodes of nonbilious, nonbloody vomiting, and diarrhea. She reported no known food allergies and denied the use of herbal products, over-the-counter supplements, tobacco, alcohol, or illicit drugs.

The patient’s home medication regimen included IV piperacillin-tazobactam 3.375 g every eight hours and IV vancomycin 1 g every 12 hours, both initiated five weeks prior to presentation. Additional medications included amitriptyline 10 mg nightly, celecoxib 200 mg daily as needed, fluoxetine 40 mg nightly, hydrocodone-acetaminophen 7.5/325 mg every six hours as needed, levetiracetam 500 mg twice daily, ondansetron 4 mg as needed, ropinirole 2 mg at bedtime, ezetimibe 10 mg daily, and rosuvastatin 10 mg nightly.

Clinical findings

On examination, the patient was afebrile with a temperature of 36.8°C, blood pressure of 124/82 mmHg, heart rate of 106 beats per minute, respiratory rate of 18 breaths per minute, and oxygen saturation of 97% on room air. She was alert and oriented to time, place, and person and exhibited no focal sensory or motor deficits. Her abdomen was soft, non-tender, and non-distended, with normal bowel sounds. Cardiovascular assessment showed a regular rhythm without murmurs. Lung examination revealed clear breath sounds bilaterally, with no wheezes, rales, or rhonchi. Skin evaluation showed no petechiae or ecchymoses.

Diagnostic assessment

Laboratory results on admission are demonstrated in Tables 1-2.

**Table 1:** Laboratory data obtained on admission * abnormal laboratory values BUN: blood urea nitrogen, AST: aspartate aminotransferase, ALT: alanine aminotransferase, ALP: alkaline phosphatase, TSH: thyroid-stimulating hormone, A1C: glycated hemoglobin, INR: international normalized ratio

Parameters	Patient's values on admission	Reference range, adults
Hemoglobin (g/dL)	12	12.0-15.5
Hematocrit (%)	36.6	34.9-44.5
White cell count (per mm3)	6,800	3,500-10,500
Platelet count (per mm3)	119,000*	150,000-450,000
Sodium (mEq/dL)	136	135-145
Potassium (mEq/dL)	3.4	3.5-5.1
Bicarbonate (mEq/dL)	25	22-29
BUN (mg/dL)	8	12-21
Creatinine (mg/dL)	1.35*	0.55-1.02
AST (units/L)	58*	12-31
ALT (units/L)	97*	9-29
ALP (units/L)	110	40-150
Total bilirubin (mg/dL)	0.2	0.2-1.2
Magnesium (mg/dL)	1.4*	1.6-2.6
Phosphorus (mg/dL)	2.0*	2.3-4.7
Lactate (mmol/L)	1.2	0.9-1.7
TSH (mcIU/mL)	1.451	0.35-4.95
A1C	5.2	≤6.5
INR	1	-

**Table 2:** Summary of urinalysis, urine microscopy, and toxicology findings

Test category	Parameter	Patient result	Reference range
Urinalysis	Color	Yellow	Yellow to straw-colored
	Protein	100 mg/dL	Negative to trace
	Glucose	Negative	Negative
	Ketones	Negative	Negative
	Blood	Negative	Negative
	Leukocyte esterase	Negative	Negative
	Nitrites	Negative	Negative
	pH	6.0	4.5-8.0
	Specific gravity	1.010	1.005-1.030
Urine microscopy	RBCs	<2	0-3 per HPF
	WBCs	<2	0-5 per HPF
	Eosinophils	8%	0% (none expected)
	Casts	None	None or occasional hyaline casts
	Crystals	None	None or occasional (varies)
Urine toxicology screen	Benzodiazepines	Negative	Negative
	Methamphetamines	Negative	Negative
	Cocaine	Negative	Negative
	Opiates	Negative	Negative
	Phencyclidine (PCP)	Negative	Negative
	Methadone	Negative	Negative
	Cannabinoids	Negative	Negative
	Barbiturates	Negative	Negative

**Figure 1:**
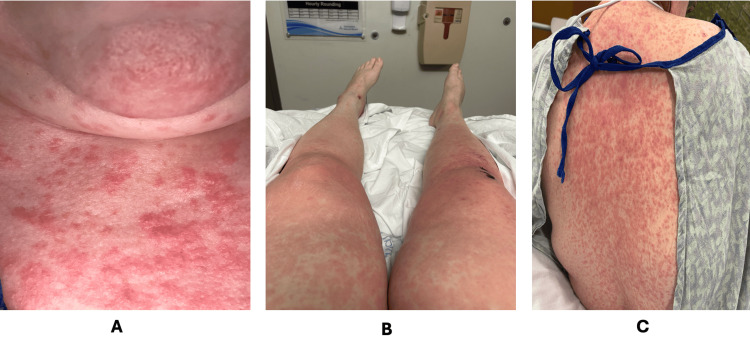
Widespread morbilliform erythematous rash affecting the chest (A), lower extremities (B), and upper back (C) in the setting of acute DRESS syndrome DRESS: drug reaction with eosinophilia and systemic symptoms

The patient was initially managed with IV fluids and electrolyte repletion, including potassium, magnesium, and phosphorus. Her nausea, vomiting, and diarrhea were treated symptomatically with antiemetic and antimotility agents. Her IV antibiotics were switched to oral doxycycline 100 mg twice daily. On hospital day 2, she developed a high-grade fever, reaching 39.5°C, accompanied by a sore throat and the onset of an intensely pruritic, generalized morbilliform rash (Figure 1).

An extensive infectious evaluation was conducted to determine the cause of the patient’s fever, rash, and elevated liver enzymes (Table 3). Blood cultures were obtained, and the peripherally inserted central catheter was removed; both the catheter tip and blood cultures ultimately showed no growth. A repeat urinalysis revealed proteinuria accompanied by eosinophils on urine microscopy, raising concern for interstitial nephritis. Additional infectious studies, including respiratory viral testing, hepatitis serologies, and human immunodeficiency virus (HIV) antigen screening, were likewise negative. Stool studies, including culture, fecal leukocyte analysis, and ova and parasite examination, were unremarkable, and testing for *Clostridioides difficile* was negative. EBV serologies were also negative, effectively ruling out acute mononucleosis as the source of her fever, transaminitis, and morbilliform eruption. The comprehensive negative infectious workup supported a noninfectious etiology for her presentation. Furthermore, toxic and autoimmune causes of liver injury were excluded based on the laboratory findings summarized in Table 3.

**Table 3:** Additional comprehensive diagnostic laboratory workup Hep A IgM: hepatitis A virus immunoglobulin M; Hep B sAg: hepatitis B virus surface antigen; Hep C Ab: hepatitis C virus antibody; Hep B core IgM: hepatitis B virus core antibody immunoglobulin M; HIV Ag, Ab: human immunodeficiency virus antigen, antibody; RSV: respiratory syncytial virus by nucleic acid amplification test; ANA: antinuclear antibody; ASMA: anti-smooth muscle antibody; AMA: antimitochondrial antibody; CMV: cytomegalovirus; EBV: Epstein-Barr virus

Parameters	Patient's values	Reference range, adults
	Infectious	
Hep A IgM	Nonreactive	-
Hep Bs Ag	Nonreactive	-
Hep C Ab	Nonreactive	-
Hep B core IgM	Nonreactive	-
HIV Ag, Ab combo screen	Nonreactive	-
Herpes simplex virus	Negative	-
EBV	Negative	-
CMV	Negative	-
Rubeola IgG	Negative	-
Influenza A	Negative	-
Influenza B	Negative	-
RSV by NAAT	Negative	-
Covid 19	Negative	-
Group A *Streptococcus* by NAAT	Negative	-
Infectious mononucleosis screen	Negative	-
	Toxic	
Acetaminophen level (mcg/mL)	<3.0	≤30.0
Salicylate level (mg/dL)	<5.0	5-29
Ethanol (mg/dL)	<10	12-21
	Autoimmune	
ANA	Negative	-
ASMA (units)	7	0-19
AMA (u/mL)	1.3	≤3.9

**Figure 2:**
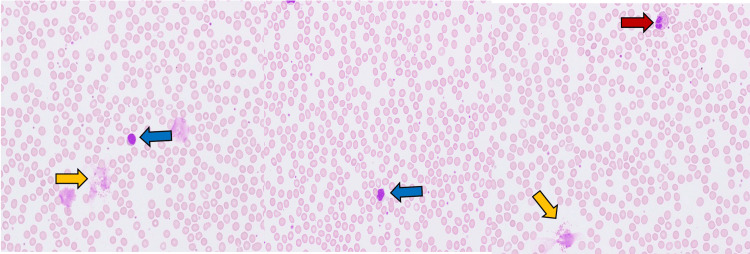
Peripheral blood smear demonstrating eosinophils (red arrow), smudged eosinophils (yellow arrows), and atypical lymphocytes (blue arrows)

**Figure 3:**
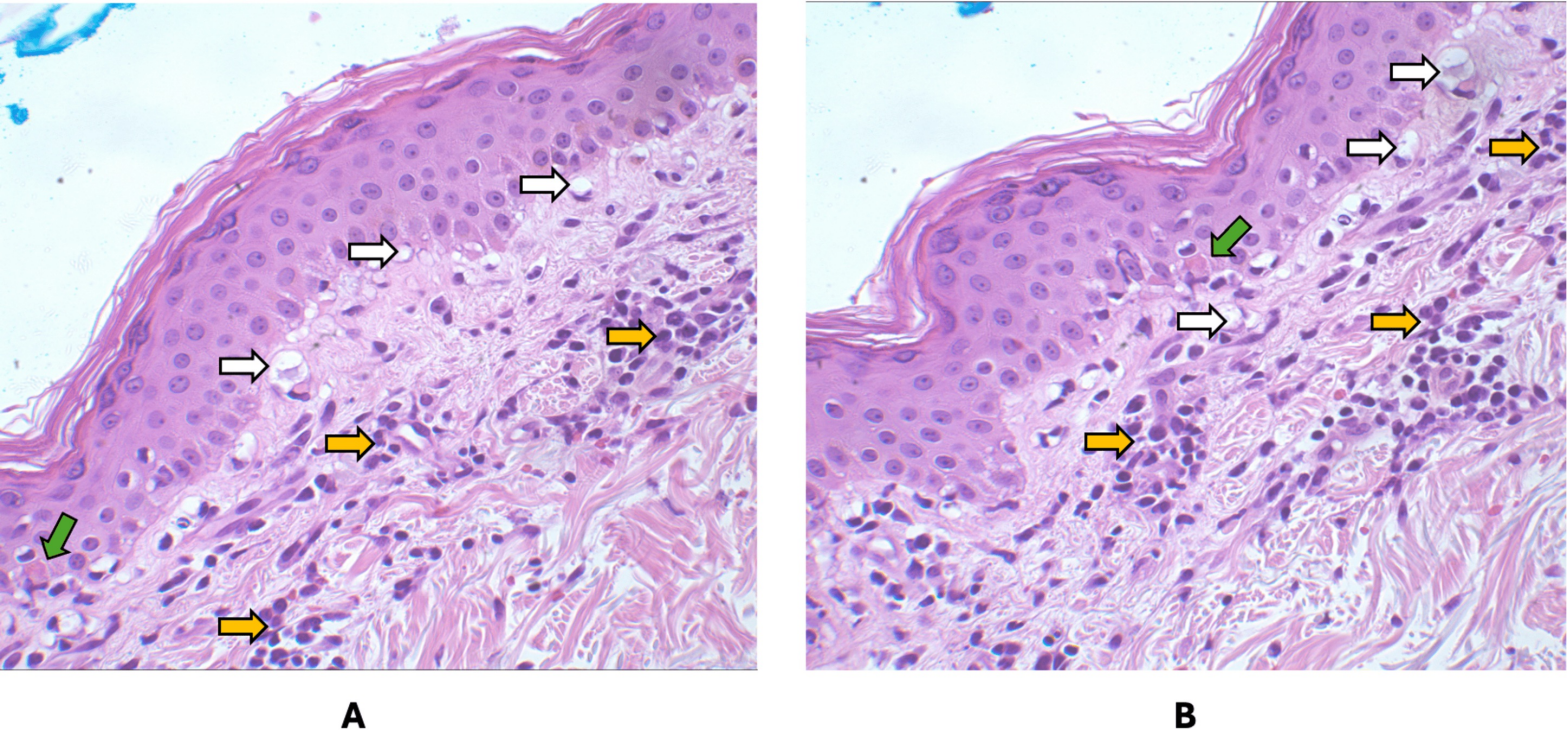
Skin biopsy findings in DRESS syndrome H&E-stained sections demonstrate interface dermatitis, characterized by basal vacuolar degeneration (white arrows), dense lymphocytic infiltration within the superficial dermis and along the dermoepidermal junction (yellow arrows), and scattered dyskeratotic keratinocytes (green arrows), consistent with a drug-induced hypersensitivity reaction. H&E: hematoxylin and eosin, DRESS: drug reaction with eosinophilia and systemic symptoms

On hospital day 4, the patient developed new-onset eosinophilia with an absolute eosinophil count of 950 cells/µL, which increased to 1,230 cells/µL (reference range 0-700 cells/µL) on day 5 and was accompanied by atypical lymphocytes on peripheral smear (Figure 2; Table 4). In response to these findings, doxycycline was discontinued due to concern for a drug-induced hypersensitivity reaction. Given her prior exposure to minocycline during a previous hospitalization and subsequent treatment with vancomycin after discharge, clinical suspicion for DRESS syndrome was high. Based on the RegiSCAR criteria, she received an initial score of 5, supporting a diagnosis of probable DRESS. IV dexamethasone 10 mg daily was initiated for three days, followed by a skin biopsy. Histopathologic evaluation revealed interface dermatitis with lymphocytic infiltration and scattered dyskeratotic keratinocytes, findings characteristic of DRESS syndrome (Figure 3). With these histopathologic findings and the resolution of her symptoms after more than 15 days, her RegiSCAR score increased to 7, indicating a definite diagnosis of DRESS syndrome.

**Table 4:** Laboratory trends throughout hospitalization * abnormal laboratory values ALT: alanine aminotransferase, AST: aspartate aminotransferase, ALP: alkaline phosphatase

Parameters	Patient's values on day 2	Patient's values on day 3	Patient's values on day 4	Patient's values on day 5	Patient's values on day 6 (after steroids initiation)	Patient's values on day 9	Patient's values on day 10	Reference range, adults
Hemoglobin (g/dL)	10.8*	10.9*	11.6*	11.7*	9.5*	9.6*	9.6*	12.0-15.5
									Hematocrit (%)	32.7*	32.4*	37.1	35.4	28.5*	28.9*	29.2*	34.9-44.5
White cell count (per mm3)	6,100	8,100	9,500	10,600	11,000	11,500*	12,800*	3,500-10,500									
									Platelet count (per mm3)	160,000	181,00	227,000	242,000	258,000	377,000	408,000	150,000-450,000
Eosinophils absolute count (K/uL)	0.1	0.4	0.95*	1.23*	0	0.1	0.12	0.0-0.7									
									Basophilis absolute count (K/uL)	0	0	0.11*	0.12*	0.11*	0.24*	0.34*	0.0-0.1
Lymphocytes absolute count (K/uL)	0.24*	0.35*	1.17	0.45*	1.98	1.5	2.95	0.45-4.8									
									Neutrophils absolute count (K/uL)	6.34	7.29	8.16	9.52*	8.47	8.36	8.97*	1.6-8.5
Monocytes absolute count (K/uL)	0.16	0.32	0	0.86*	0.33	0.66	0.58	0.0-0.8									
									ALT (units/L)	47*	44*	41*	31*	28	389*	365*	9-29
AST (units/L)	66*	62*	48*	43*	46*	474*	311*	12-31									
									ALP (units/L)	90	83	85	82	79	104	114	40-150
Creatinine (mg/dL)	1.2*	1.26*	1.09*	1.24*	1.09*	0.99	1	0.55-1.02									

**Figure 4:**
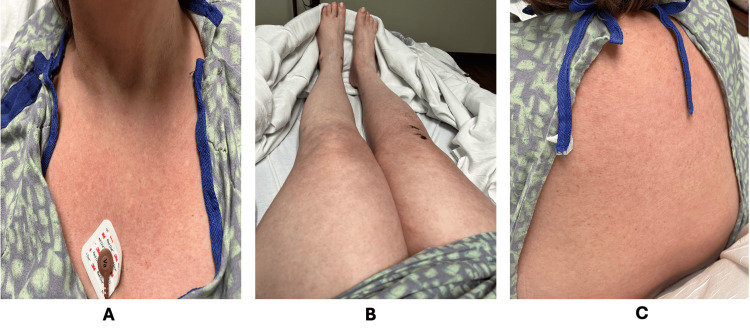
Marked improvement of morbilliform eruption following systemic corticosteroid therapy, demonstrated on the chest (A), lower extremities (B), and upper back (C)

Following initiation of corticosteroid therapy, the patient’s fever resolved, her rash progressively improved (Figure 4), renal function returned to baseline, and liver enzymes transiently elevated before stabilizing (Table 4). Intravenous corticosteroids were subsequently transitioned to oral prednisone at a dose of 60 mg daily, with a planned six-week taper. At a one-week follow-up, hepatic transaminases had normalized (ALT 26 units/L, AST 31 units/L), consistent with complete resolution of DRESS syndrome. A detailed overview of the clinical timeline is outlined in Table 5.

**Table 5:** Chronological overview of patient’s clinical progression IV: intravenous, GI: gastrointestinal, AKI: acute kidney injury, DRESS: drug reaction with eosinophilia and systemic symptoms, LFTs: liver function tests

Timeline	Event	Details
5 weeks before admission	Initial antibiotic exposure	Received IV minocycline during hospitalization for osteomyelitis
Post-discharge	Home antibiotic therapy	Continued IV vancomycin + IV piperacillin-tazobactam
Day 0 (infusion center)	Syncopal episode	Occurred during IV vancomycin infusion
Day 1 (admission)	GI symptoms	Nausea, vomiting, diarrhea; mild AKI, mild transaminitis
Day 1	Antibiotic change	IV antibiotics discontinued → Switched to oral doxycycline
Day 2	New fever + morbilliform rash	Tmax 39.5°C; rash rapidly spreading
Day 3	Rash progression	No eosinophilia yet
Day 4	Eosinophilia begins	Absolute eosinophils 950/µL, doxycycline stopped due to concern for drug reaction
Day 5	Peak symptoms	Fever + worsening rash; eosinophils 1,230/µL; atypical lymphocytes
	Treatment initiated BEFORE biopsy	IV dexamethasone 10 mg daily started due to high clinical suspicion for DRESS
	Skin biopsy obtained	Histology later confirmed DRESS
Day 6–8	Clinical improvement	Fever resolved and rash improved on steroids
Day 10 (discharge)	Labs improving	Liver function tests demonstrated a transient elevation, followed by a plateau, while serum creatinine trended toward baseline.
1-week follow-up	Resolution	LFTs normalized; rash nearly resolved; prednisone taper ongoing

## Discussion

DRESS syndrome represents a severe, delayed hypersensitivity reaction with multisystem involvement, posing significant diagnostic and therapeutic challenges [1,2]. The concurrent use of vancomycin and minocycline in this patient illustrates the complexity of identifying causative agents, as both antibiotics have been increasingly linked to drug-induced hypersensitivity reactions [4,5]. The overlapping clinical features with infectious, autoimmune, or other dermatologic conditions often complicate timely recognition, emphasizing the necessity for a meticulous medication history and systematic evaluation [1,6].

The diagnosis of DRESS syndrome is primarily clinical and can be challenging due to its heterogeneous presentation and delayed onset. The RegiSCAR scoring system provides a structured approach to evaluate suspected cases, incorporating criteria such as timing of drug exposure, type and extent of skin eruption, presence of fever, lymphadenopathy, hematologic abnormalities (eosinophilia and atypical lymphocytes), and objective evidence of internal organ involvement (Table 6) [2,8].

**Table 6:** RegiSCAR scoring system for the diagnosis of DRESS syndrome The score can be calculated online via the MDCalc tool [8]. RegiSCAR: Registry of Severe Cutaneous Adverse Reactions, DRESS: drug reaction with eosinophilia and systemic symptoms

Variable		Points
Fever (≥38.5°C)	No/unknown	-1
	Yes	0
Enlarged lymph nodes (≥2 sites, >1 cm)	No/unknown	0
	Yes	1
Atypical lymphocytes	No/unknown	0
	Yes	1
Eosinophilia	0-699 cells or <10% (no eosinophilia)	0
	700-1,499 cells or 10-19.9%	1
	≥1,500 cells or ≥20%	2
Skin rash extent >50%	No/unknown	0
	Yes	1
At least two of: edema, infiltration, purpura, scaling	No	-1
	Yes	1
	Unknown	0
Biopsy suggesting DRESS	No	-1
	Yes/unknown	0
Internal organ involved	0	0
	1	1
	≥2	2
Resolution in ≥15 days	No/unknown	-1
	Yes	0
Alternative diagnoses excluded (by ≥3 biological investigations)	No/unknown	0
	Yes	1

Each criterion is assigned a score, and the cumulative total stratifies cases into “no,” “possible,” “probable,” or “definite” DRESS (Table 7), facilitating standardized diagnosis and comparison across studies. Histopathologic findings can support the diagnosis but are not definitive, emphasizing the importance of integrating clinical, laboratory, and medication history data in the diagnostic process [2]. The RegiSCAR score has been validated in multicenter studies and is widely used in both clinical practice and research to improve diagnostic accuracy and guide timely management [2].

**Table 7:** Interpretation of the RegiSCAR diagnostic scoring system RegiSCAR: Registry of Severe Cutaneous Adverse Reactions, DRESS: drug reaction with eosinophilia and systemic symptoms

RegiSCAR-group score	Likelihood of DRESS diagnosis
<2	No case
2-3	Possible case
4-5	Probable case
>5	Definite case

According to the RegiSCAR scoring system, the patient received an initial score of 5, which meets the criteria for probable DRESS syndrome. Her initial score was calculated as follows: 0 points for fever ≥38.5°C; 0 points for lymphadenopathy; +1 for atypical lymphocytes; +1 for eosinophilia; +1 for a skin eruption involving more than 50% of the body surface area; +1 for edema, purpura, and scaling; −1 for skin biopsy findings not supportive of DRESS; +2 for involvement of two internal organs (liver and kidney); −1 for clinical resolution exceeding 15 days; and +1 for adequate exclusion of alternative diagnoses. Following receipt of the skin biopsy results and initiation of corticosteroid therapy, her RegiSCAR score increased to 7, consistent with a definite case of DRESS syndrome. This reassessment incorporated the same criteria as above, with updated scores of 0 for biopsy findings (no longer negative) and 0 for time to clinical resolution.

Hepatic and renal dysfunction are among the most clinically significant organ manifestations of DRESS syndrome and play a major role in determining overall prognosis [9]. Hepatic involvement is widespread, occurring in approximately 50% to 87% of cases [11]. The extent of liver injury varies widely, ranging from mild transaminase elevations to fulminant hepatic failure requiring transplantation [11]. A retrospective cohort study conducted in Taiwan using a national DRESS registry and published in the Journal of the American Academy of Dermatology reviewed 72 confirmed cases. Liver involvement was identified in 62 patients (86.1%). Among these, cholestatic injury was the most common pattern at initial presentation (23 patients, 37.1%), followed by mixed (17 patients, 27.4%) and hepatocellular injury (12 patients, 19.4%) [9]. Our patient’s initial R-factor was 2.2, consistent with a mixed pattern of liver injury, which increased to 7.8 by the time of discharge, indicating a transition to a hepatocellular pattern. Recovery time is likewise variable and often correlates with the initial severity of hepatocellular injury; patients with markedly elevated transaminases (e.g., >10× the upper limit of normal) may require several weeks to months for full biochemical recovery [9].

Renal involvement is observed in approximately 10-30% of cases. It most commonly manifests as acute interstitial nephritis (AIN), typically presenting with AKI and associated mild proteinuria, hematuria, and, at times, eosinophiluria, as demonstrated in our patient. In a systematic review of 71 biopsy-confirmed cases, 96% of patients developed AKI, with AIN identified as the predominant histopathologic finding. Notably, most patients recovered renal function with timely recognition and appropriate therapy [12].

Hematologic abnormalities, including eosinophilia and atypical lymphocytosis, further substantiated the diagnosis and mirrored underlying T-cell-mediated immune dysregulation [1,2]. Early identification of these laboratory markers can help differentiate DRESS from other mimicking syndromes.

Prompt cessation of the offending drugs is essential to halt progression, and initiation of systemic corticosteroids remains the mainstay for controlling organ involvement [10]. The patient demonstrated a favorable response to corticosteroid therapy, highlighting its efficacy in moderating inflammatory processes and preventing permanent organ injury. Close monitoring during therapy and gradual tapering are crucial to detect potential relapse or late-onset complications [10].

Recognition of antibiotics as emerging triggers of DRESS necessitates vigilance among clinicians, particularly when patients present with fever, rash, and organ dysfunction several weeks after initiating therapy. Detailed documentation and reporting of such occurrences will enhance understanding of disease patterns, improve early recognition, and refine management strategies.

## Conclusions

DRESS syndrome remains a diagnostically challenging and potentially life-threatening adverse drug reaction, particularly when triggered by multiple concurrent medications such as vancomycin and minocycline. Its variable presentation, delayed onset, and overlap with infectious or autoimmune conditions often contribute to delayed recognition. This case underscores the importance of maintaining a high index of suspicion in patients who develop fever, rash, eosinophilia, and organ dysfunction weeks after exposure to high-risk drugs. Early identification, prompt withdrawal of the offending agents, and timely initiation of systemic corticosteroids were critical in preventing further hepatic and renal deterioration in this patient. As antibiotic-associated DRESS becomes increasingly recognized, clinicians must be aware of its diverse manifestations and the need for close multidisciplinary monitoring. Continued reporting of such cases will help refine diagnostic approaches and guide evidence-based management strategies.
